# Identifying optimal capsid duplication length for the stability of reporter flaviviruses

**DOI:** 10.1080/22221751.2020.1829994

**Published:** 2020-10-14

**Authors:** Coleman Baker, Yang Liu, Jing Zou, Antonio Muruato, Xuping Xie, Pei-Yong Shi

**Affiliations:** a Departement of Microbiology and Immunology, University of Texas Medical Branch, Galveston, Texas, USA; b Department of Biology and Molecular Biology, University of Texas Medical Branch, Galveston, Texas, USA; c Department of Pharmacology and Toxicology, University of Texas Medical Branch, Galveston, TX, USA; d Sealy Center for Structural Biology and Molecular Biophysics, University of Texas Medical Branch, Galveston, TX, USA; e Sealy Institute for Vaccine Sciences, University of Texas Medical Branch, Galveston, TX, USA; f Sealy Institute for Translational Sciences, University of Texas Medical Branch, Galveston, TX, USA

**Keywords:** Flavivirus, reporter virus, recombination, replication, capsid

## Abstract

Mosquito-transmitted flaviviruses cause widespread disease across the world. To provide better molecular tools for drug screens and pathogenesis studies, we report a new approach to produce stable NanoLuc-tagged flaviviruses, including dengue virus serotypes 1-4, Japanese encephalitis virus, yellow fever virus, West Nile virus, and Zika virus. Since the reporter gene is often engineered at the capsid gene region, the capsid sequence must be duplicated to flank the reporter gene; such capsid duplication is essential for viral replication. The conventional approach for stabilizing reporter flaviviruses has been to shorten or modify the duplicated capsid sequence to minimize homologous recombination. No study has examined the effects of capsid duplication length on reporter virus stability. Here we report an optimal length to stabilize reporter flaviviruses. These viruses were stable after ten rounds of cell culture passaging, and in the case of stable NanoLuc-tagged Zika virus (ZIKV C38), the virus replicated to 10^7^ FFU/ml in cell culture and produced robust luciferase signal after inoculation in mosquitoes. Mechanistically, the optimal length of capsid duplication may contain all the *cis*-acting RNA elements required for viral RNA replication, thus reducing the selection pressure for recombination. Together, these data describe an improved method of constructing optimal reporter flaviviruses.

## Introduction

Viruses from the arthropod-borne genus Flavivirus afflict people across the globe causing febrile, neurologic, and hemorrhagic disease [1]. Notable among the flaviviruses are the four serotypes of dengue virus (DENV), which cause an estimated 96 million symptomatic infections yearly [2], Japanese encephalitis virus (JEV) which causes the annual loss of 709,000 disability-adjusted life years [3], the recently emerged Zika virus (ZIKV), which has become associated with congenital malformations [4], encephalitic West Nile virus (WNV), and yellow fever virus (YFV), which periodically emerges from its sylvatic transmission cycle to start an urban transmission cycle [5]. Concerted efforts by scientists and clinicians have brought about vaccines for YFV, JEV, and DENV [6,7] though effective antiviral drugs have yet to be approved. Reporter flaviviruses, first published in 2003 [8], have been critical for high-throughput antiviral compound screens [9,10], host and virus pathogenesis studies [11], and serological diagnosis [12,13]. Despite these advances, reporter flaviviruses suffer from genetic instability during longer periods of growth or passaging, thought to be primarily mediated by recombination [14,15].

Reporter genes are routinely engineered at the beginning of the single open reading frame of the viral polyprotein, between the 5’ UTR and the capsid gene, as first described using YFV [16]. RNA signals that aid in genome cyclization, which is essential for viral replication, and facilitate translation are continuous from the 5’ UTR into the beginning of the capsid. These signals must function together, therefore it is necessary to duplicate a portion of the capsid gene and place it upstream of the inserted reporter gene. Until recently, efforts to stabilize these constructs centered on reducing homology between the duplicated capsid sequences by codon scrambling in an effort to reduce homologous recombination [16,17]. This also had the benefit of expunging the *cis-*acting elements in the full capsid sequence, leaving only the upstream elements. Two additional methods for stabilizing reporter flaviviruses have been newly developed, both focusing on blocking recombined reporter viruses from continued infection. Volkova and colleagues report a single-nucleotide insertion to the duplicated capsid portion of a reporter ZIKV that minimally perturbs critical RNA elements but causes a +1 frameshift [18]. If recombination occurs between the duplicated capsid sequences that flank the reporter gene, this frameshift mutation is incorporated into the viral polyprotein and causes mistranslation, effectively taking out recombined viruses from the population. We developed a related method for reporter ZIKV and YFV using recombination-dependent lethal mutations in the duplicated capsid [19]. These lethal mutations stop viral particle formation if recombination brings them into the viral polyprotein.

The length of capsid duplications in different flavivirus reporter constructs that have been reported varies from 25, to 33 or 34, 38, 50, or even the full capsid [10,16–18,20–24.] Shorter lengths are tolerated by some viruses and not by others. At the onset of this investigation, there was no published comparison of the effect of capsid duplication length and its effect on stability. It was believed that shorter capsid repeats were preferred because the shorter homologous sequence minimizes homologous recombination. We hypothesize that an optimal length of capsid duplication is required for efficient viral replication; a shortened capsid duplication imposes a selection pressure on viral replication, leading to undesired recombination and deletion of the engineered reporter gene. The goal of this study is to test this hypothesis by engineering different lengths of capsid duplication and investigating the length effect on the stability of the reporter gene in various flaviviruses. Indeed, we found an optimal length of capsid duplication of 34 or 38 amino acids that can increase the reporter gene stability for at least ten rounds of cell culture passages. Taking this new approach, we have developed a panel of long-term stable NanoLuc-tagged flaviviruses, including the four serotypes of DENV, JEV, YFV, WNV, and ZIKV. In addition, we demonstrated the use of the reporter flaviviruses for rapid antibody neutralization testing and antiviral drug discovery. Taken together, our results have established a previously unrecognized approach to generate stable reporter flaviviruses that are useful for research and countermeasure development.

## Materials and methods

### Viruses and cells

Zika virus strain Dakar 41525, yellow fever 17D strain YFS11, dengue virus 1 strain Western Pacific, dengue virus 2 strain New Guinea C, Japanese encephalitis virus 14-14-2 strain 1454, and West Nile virus strain NY99 with NS5 E218A [25,26] were cloned into full-length plasmids using the low copy pCC1 vector as has been previously described [27]. NEBuilder HiFi DNA Assembly mix (NEB E2621) was used to assemble all plasmids. Full length dengue virus 3 strain VN32 and dengue virus 4 strain MY01 transcripts were assembled from four and three fragments, respectively, by *in* vitro ligation. Viruses were recovered after electroporation (Biorad GenePulser Xcell) of *in vitro* transcribed RNAs in Vero (ATCC Cat# CCL-81) or BHK21 (ATCC Cat# CCL-10) cells as previously described [20]. Vero and BHK21 cells were grown in Dulbecco’s Modified Eagle Medium (Gibco 11965) with 10% fetal bovine serum (FBS, Hyclone SH30071) and 1% penicillin/streptomycin (Gibco 15140). Huh7 cells were grown in Dulbecco’s Modified Eagle Medium with Glutamax (Gibco 10566) supplemented with 10% FBS, 1% penicillin/streptomycin, and 1% non-essential amino acids (Gibco 11140). Infections were carried out in the same media excepting supplementation with 2% fetal bovine serum instead of 10%. Vero, BHK21, and Huh7 cells were grown at 37°C in a humidified incubator with 5% CO_2_.

### Immunofluorescence assay

Vero cells were seeded into chamber slides immediately post-electroporation. At the indicated time points, cells were washed with PBS and fixed with cold methanol at −30°C for >30 minutes. Slides were washed with PBS and blocked with PBS+1% FBS overnight at 4°C. The flavivirus envelope reactive antibody 4G2 (ATCC Cat# HB-112), diluted in blocking buffer, was used as a primary antibody incubated for 1 hour at room temperature. A secondary goat anti-mouse IgG antibody conjugated with Alexa Fluor 488 (Thermo Fisher Scientific Cat# A-11001) was then used to probe for 4G2 for 1 hour. Slides were then washed 3X with PBS and stained with DAPI (Vector Laboratories, H-1200) and then imaged on a Nikon Eclipse Ti2 microscope. ImageJ (NIH) was used to process the images.

### Focus forming assay

All viruses were titered by focus-forming assay. Viruses were serially diluted ten-fold in DMEM supplemented with 2% FBS and used to infect Vero cells that had been seeded the day previously at 2 × 10^5^ cells per well in a 24-well plate. After a 1-h infection with rocking every 15 minutes, the virus was removed and methylcellulose/DMEM overlay was added. After four days of infection, the overlay was removed, and cells were fixed with a 1:1 solution of methanol/acetone for >15 minutes. Plates were then washed 3X with PBS, blocked with PBS+3% FBS for 30 minutes, and then incubated with virus-specific mouse immune ascites fluid (MIAF, World Reference Center for Emerging Viruses and Arboviruses, UTMB). After >1-h incubation with MIAF, plates were washed with PBS and incubated with a horseradish peroxidase-conjugated anti-mouse IgG antibody (SeraCare KPL Cat# 474-1806). After a 3X PBS wash, foci were developed in the dark using an AEC peroxidase substrate kit (Enzo 43825) according to the manufacturer’s protocol and imaged using a BioRad ChemiDoc Imaging System.

### Reporter virus passaging and stability

Virus recovered from electroporation was termed P0. 500 µL of this was added to a T75 flask with a confluent layer of Vero cells. The infection was discontinued once cell death was observed after which media was harvested. 500 µL of the new passage was then used to infect a new T75 flask for the next passage. This was carried out in parallel series for each virus. Stability was assessed by isolating viral RNA (Qiagen 52904 or TriZOL, Invitrogen 15596026) from each passage and using this as a template for an RT-PCR reaction (Invitrogen 12574) with primers that spanned the 5’ UTR to the end of the capsid. The products were then run on a 0.6% agarose gel and imaged with a BioRad GelDoc EZ Imager.

### Growth kinetics by focus forming assay

Six well plates were seeded at 8 × 10^5^ cells per well with Vero cells the day before infection. Cells were infected at a MOI of 0.01 in triplicate for 1 h with shaking every 15 minutes, followed by a 3X PBS wash and addition of media supplemented with 2% FBS. Supernatant samples were taken at 24, 48, 72, 96, and 120 h and titered by focus-forming assay.

### Reporter neutralization assays

Reporter neutralization tests were done by two-fold serially diluting sera, starting at 1:50 in DMEM with 2% FBS. Sera samples positive for ZIKV and DENV1-4 were pooled from mice infected with the respective virus. YFV and JEV sera samples were from vaccinated mice. Sera dilutions were mixed 1:1 by volume with the respective reporter virus and incubated at 37°C for 1 h. The virus/sera mixture was then plated on Vero cells in a white, opaque 96 well plate that was seeded at 1.5 × 10^4^ cells per well. After a 4-h incubation at 37°C, the wells were washed 2X with PBS and 50 µL of NanoGlo substrate diluted 1:50 in NanoGlo Assay Buffer was added to the cells. Plates were read in a BioTek Cytation 5 plate reader after 3 minutes with a gain of 150. Positive controls consisted of virus infection with no sera. Negative controls comprised virus plated in wells with no cells. This negative control allows for subtraction of background luciferase signals from the virus media. After subtraction of negative controls, values were converted to a percent of the positive control. The data was then analyzed by four parameter nonlinear regression, with the top and bottom constrained to 100 and zero, respectively, and the NT_50_ reported.

### Antiviral assays

The panflavivirus inhibitor NITD008 was diluted in 90% DMSO to 10 µM and then two-fold serially diluted 8 times. These dilutions were mixed with virus (MOI 0.1-DENV1, DENV2, DENV3; MOI 0.01-ZIKV; MOI 0.001-DENV4, YFV, JEV) and plated on Huh7 cells that were seeded at 1.5 × 10^4^ cells per well the previous day in media with 2% FBS. Cells were washed 48 h post infection three times with PBS followed by addition of NanoGlo substrate diluted 1:100 in NanoGlo Assay Buffer. Plates were read by a BioTek Cytation 5 plate reader after 3 minutes.

### Mosquito infections

For the micro-injection study, Rockefeller strain *Aedes aegypti* mosquitoes were injected (100 nL) intrathoracically with virus diluted in PBS so that each mosquito received 50 FFU. Mosquitoes were cultured at 28°C for 8, 12, and 18 days. For blood feeding study, sheep’s blood was centrifuged at 1000 g, 4°C for 20 minutes to separate plasma and cells. The plasma was heat-inactivated at 56°C for 1 hour and the cells were washed twice with PBS after which they were combined again. The blood was spiked with virus to a concentration of 2×10^6^ FFU/mL. Engorged mosquitoes were further reared and harvested at 8, 12, and 18 days. At the time points indicated mosquito samples from both experiments were thoroughly homogenized (Qiagen TissueLyser II) in 200 µL PBS and centrifuged to pellet the tissue. 50 µL of supernatant was used for both RT-qPCR and luciferase assay. RNA was harvested using Qiagen RNeasy minikit and used in a Taqman RT-qPCR reaction targeting a region in NS5. *Aedes aegypti* actin served as a control. For luciferase assay, 50 µL of supernatant from homogenized mosquito was added to a 96 well opaque white plate, followed by addition of NanoGlo substrate, diluted 1:50 in NanoGlo Assay Buffer. Samples were read in a BioTek Cytation 5 plate reader. Background luciferase levels (no mosquitoes) and clean, uninfected mosquitoes were used as negative controls.

### Statistical analysis

Graphpad Prism 8 was used for graphing and statistical analysis. Statistical tests used, as well as significance levels, are noted in the figure legends. All replicated values are shown on each graph.

### Illustrations

Figures were created using Biorender and Abode Illustrator.

## Results

### Panel of reporter flaviviruses

**Figure 1.**
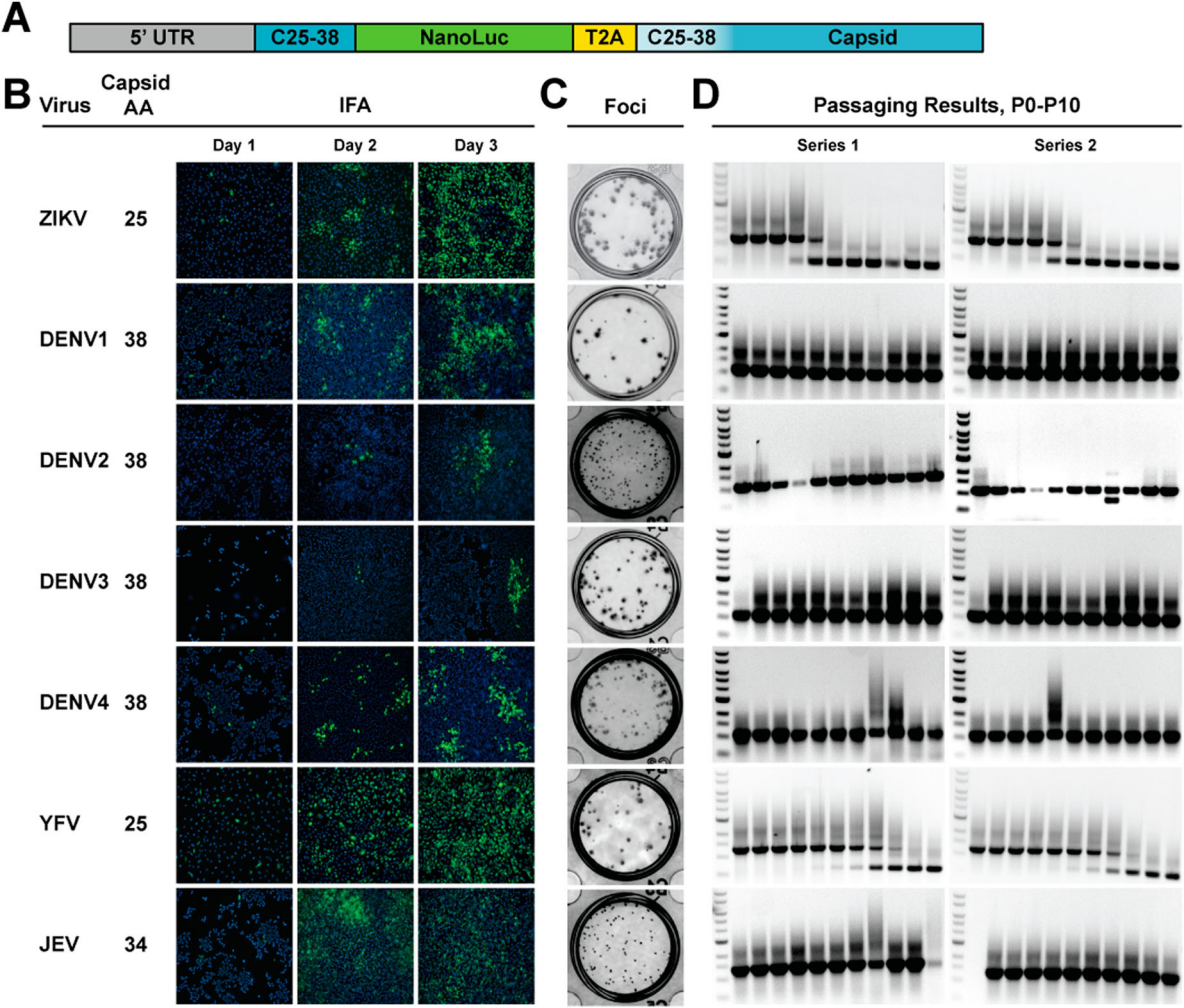
Panel of reporter flaviviruses. A. Scheme of NanoLuc insertion in the flavivirus genome. The duplicated capsid portion varies from 25 to 38 amino acids, depending on the virus. The white beginning of the capsid gene indicates the codon scrambling to reduce homology with the duplicated portion. B. Virus, corresponding number of capsid amino acids duplicated, and representative IFA images from Day 1 to 3 post electroporation. The pan-flavivirus envelope antibody 4G2 was used to probe for virus and cells were counterstained with DAPI. C. Focus-forming assay after 4-day infection on Vero cells. D. Genetic stability results after two independent series of ten passages in Vero cells. Viral RNAs from each passage were used as templates in an RT-PCR reaction with primers that spanned the 5’ UTR to the capsid. Full-length band sizes are as follows ZIKV: 1,162 bp; DENV1: 1,086 bp; DENV2: 1,128 bp; DENV3: 1,126 bp; DENV4: 1,131 bp; YFV: 1,282 bp; JEV: 1,360 bp.

Reporter flaviviruses were constructed as first described by [16] using capsid duplication lengths as found in the literature [10,16,20,23,24] (Figure 1(A,B)). All viruses except DENV3 and DENV4, which were assembled by *in vitro* ligation (Figure S1), were constructed using traditional, plasmid-based reverse genetics approaches [28]. DENV3 and DENV4 full genomes are difficult to clone in bacteria, due to putative toxic elements present [29,30]. The NanoLuc gene followed by the 2A sequence from thosea asigna virus (T2A) were inserted between a duplicated portion of the capsid and the full-length capsid. To help prevent homologous recombination, the codons in the capsid sequence corresponding to the duplicated portion were scrambled to reduce homology. All full-length DNAs were used as a template in an *in vitro* transcription reaction to generate full-length RNAs, which were subsequently electroporated into Vero cells (for ZIKV, YFV, and WNV) or baby hamster kidney (BHK) cells (for DENV1-4 and JEV). Immunofluorescence assay (IFA) was used to indicate viral spread, post electroporation (Figure 1(B) and Figure S2(A)). Focus-forming assay shows all reporter viruses form distinct foci (Figure 1(C)), but no plaques when stained with crystal violet (data not shown). The IFA and focus-forming results corroborate with the viral replication kinetics among different versions of reporter viruses (see later replication kinetic results in Figure 3). To assess stability, viruses were passaged ten times on Vero cells according to the scheme in Figure S3(A). RT–PCR products corresponding to each passage show consistent band size for all viruses out to P10, including West Nile virus (WNV) which results were obtained after the initial review of this manuscript (Figure S2(B)). The exception to this positive outcome is ZIKV and YFV, which have the shortest capsid duplication, 25 amino acids (Figure 1(D)). These two viruses showed a decrease in band size during passaging. These results suggested that the length of the capsid duplication may have an impact on virus stability.

### Extended capsid duplication

**Figure 2.**
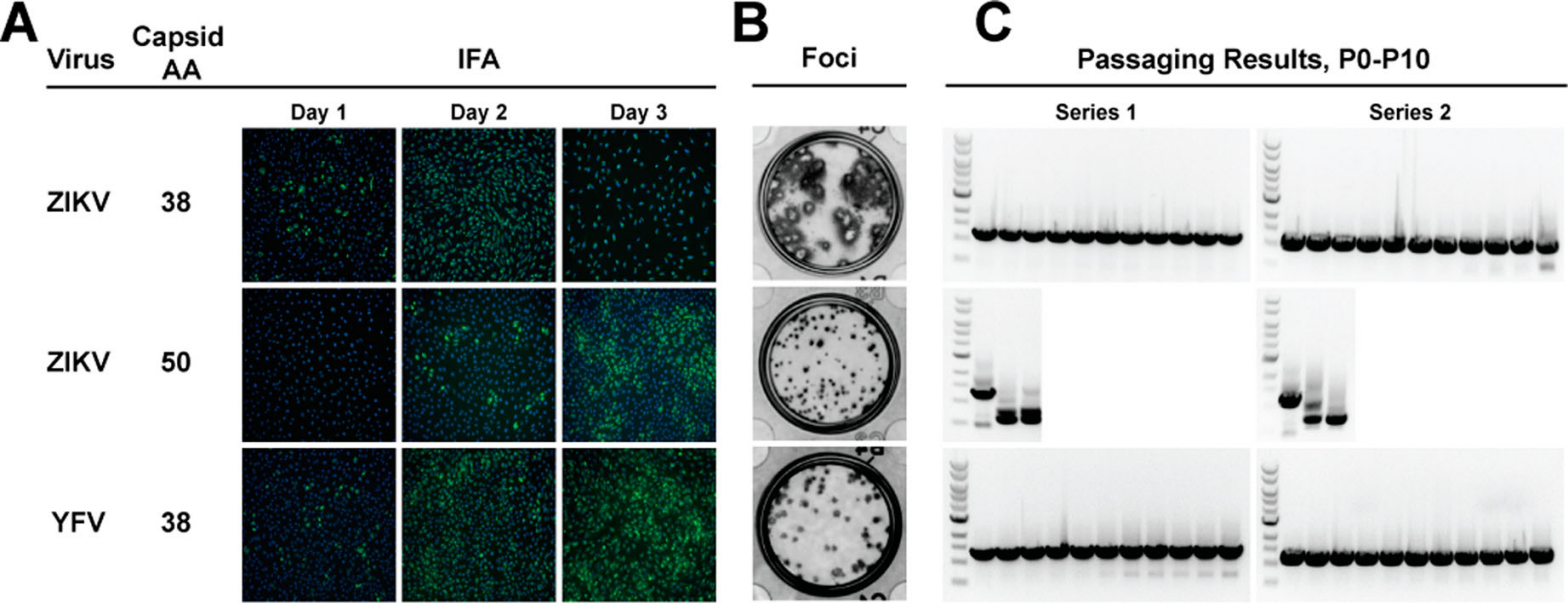
Extended capsid duplication. A. Virus, corresponding number of duplicated capsid amino acids, and representative Day 1 to Day 3 IFA images. The anti-flavivirus envelope antibody 4G2 was used. B. Focus-forming assay on Vero cells after four-day infection. C. Reporter gene retention after ten passages on Vero cells. RT-PCR products covering the reporter gene are shown for the two independent passaging series. ZIKV C50 passaging was discontinued after P2 due to loss of reporter gene. Band sizes corresponding to the full-length reporter gene are as follows ZIKV C38: 1,201 bp; ZIKV C50: 1,237 bp; YFV C38: 1,321 bp.

The results from passaging these different flaviviruses indicated that a longer capsid duplication could positively impact its stability. This hypothesis was tested by creation of ZIKV and YFV C38 NanoLuc viruses. C38 was chosen based on the robust results from DENV1-4 using this length. During this time, Volkova, et al., published a report on reporter ZIKV and the effect of capsid duplication size on replication. Their conclusion was that C50 is the optimal length for viral growth, with this being the shortest length that was not statistically attenuated compared to WT virus [18]. Based on this report, we also constructed a C50 ZIKV. IFA results post-electroporation suggested that ZIKV C38 virus replicated more robustly than the C25 and C50 virus, while YFV C38 showed little difference compared to YFV C25 (Figure 2(A), compare to Figure 1(B)). Focus size (Figure 2(B)) comparison between ZIKV C25 and ZIKV C50 showed little difference but, ZIKV C38 formed clear, larger plaques similar to non-reporter wild-type ZIKV (Figure S3(B)). YFV C38 focus size was only slightly larger than YFV C25. The C38 ZIKV and YFV and C50 ZIKV were continuously passaged and analyzed for reporter gene stability by RT–PCR (Figure 2(C)). Unexpectedly, ZIKV C50 showed early instability, so only passaging results from P0-P2 are shown. In contrast, ZIKV and YFV C38 were stable after ten rounds of continuous cell culture. These results suggest that an optimal length of duplicated capsid sequence (e.g. C38) is required for reporter virus stability. Under such condition, the frameshift or other mutations in the duplicated capsid region is not required for the stability of reporter virus.

### Effect of extended capsid duplication on viral growth

**Figure 3.**
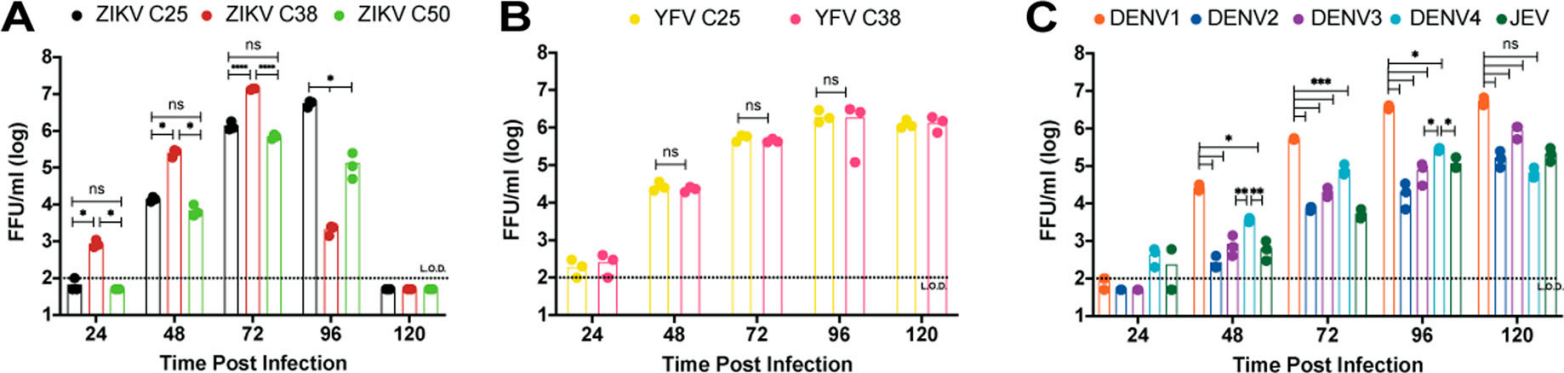
Effect of capsid duplication length on viral growth. Multi-step growth kinetics (MOI 0.01, *n* = 3) on Vero cells, using focus-forming assay to quantify growth for A. ZIKV C25, C38, and C50; B. YFV C25 and C38; C. DENV1-4 and JEV. 2-way repeated measures ANOVA with Tukey’s multiple comparisons test was used to assess significance for A and C. 2-way repeated measures ANOVA with Sidak’s multiple comparisons test was used for B (*p* > 0.5 = ns, *p* < 0.5 = *, *p* < 0.1 = **, *p* < 0.01 = ***, *p* < 0.001 = ****).

The effect of capsid duplication length on viral growth was assessed on Vero cells. Cells were infected with ZIKV C25, C38, and C50 at a MOI of 0.01 and assessed at 24, 48, 72, 96, and 120 h post infection by focus forming assay (Figure 3(A)). ZIKV C38 replicated to significantly higher titers than ZIKV C25 and ZIKV C50, reaching 10^7^ FFU/mL, at 24, 48, and 72 h post infection. ZIKV C25 and ZIKV C50 growth are similar across the same time period, though ZIKV C25 titers did continue to increase until 96 h. Conversely, growth comparison of YFV C25 and YFV C38 showed no significant difference at any time point, despite YFV C38’s increased stability (Figure 3(B)). DENV1-4 and JEV growth kinetics on Vero cells (MOI 0.01) show that DENV1 replicated significantly higher at times 24-96 h post infection (Figure 3(C)). Together these data show that among C25, C38, and C50, C38 is the most optimal capsid duplication length for ZIKV replication. In contrast to these results, there seems to be no replication advantage for YFV C38 over YFV C25.

To directly examine the effect of reporter gene insertion on viral replication, we compared the replication kinetics between the parental wild-type ZIKV and the reporter ZIKV C38 on Vero cells (Figure S3). The results showed that replication between the two is similar at alltime points but 72 h, where WT virus is 10-fold lower, possibly due to death of the host cells.

### Rapid neutralization tests and antiviral discovery

**Figure 4.**
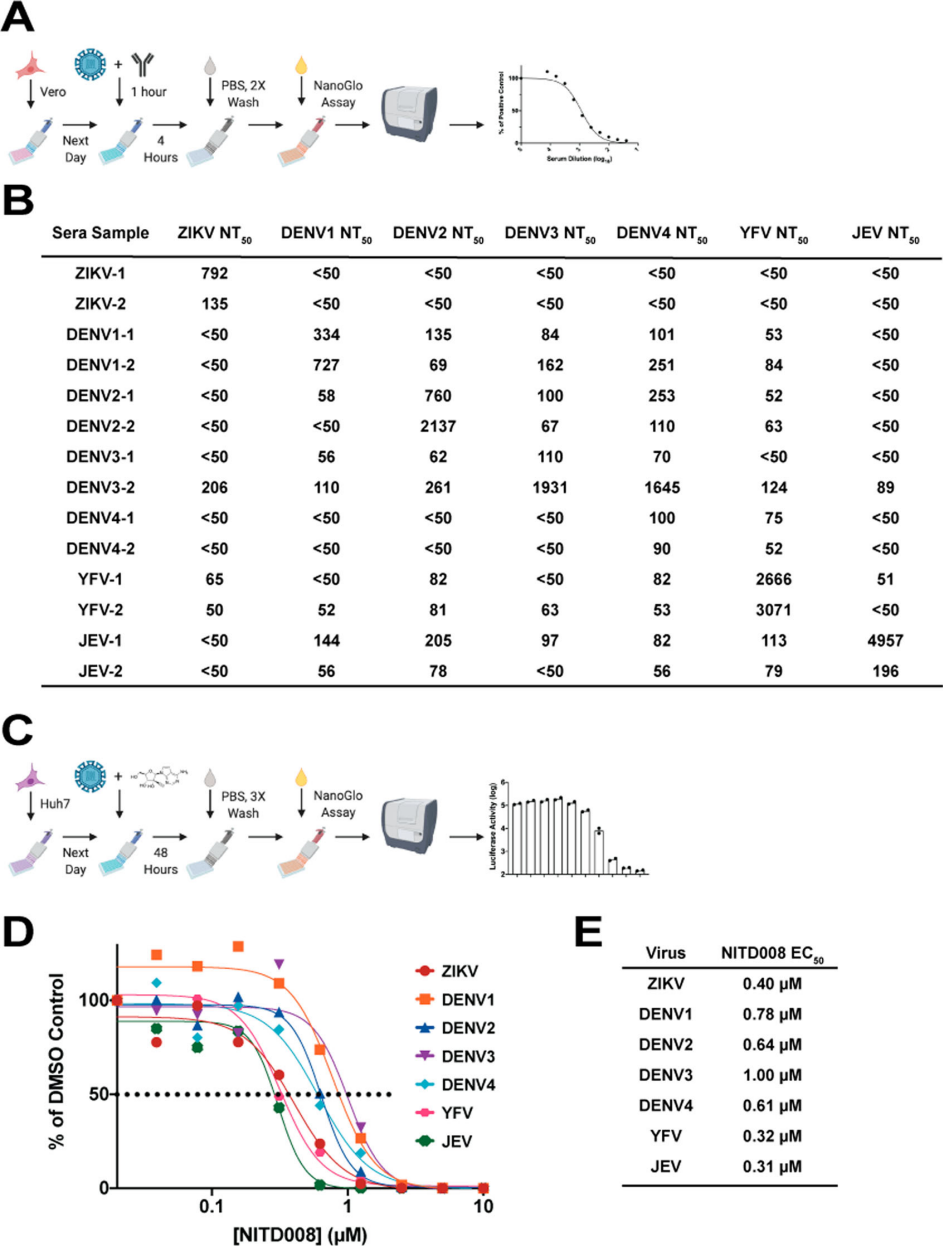
Neutralization and Antiviral Assays. A. Reporter virus neutralization scheme. Vero cells were seeded in an opaque, white 96 well plate at 1.5 × 10^4^ cells per well the day before. Sera samples were two-fold serially diluted, starting at 1:50 and then mixed with equal volume of virus and incubated for 1 h at 37°C. The sera:virus mixture was then used to inoculate Vero cells in a 96 well plate. After 4 h incubation at 37°C, plates were washed twice with PBS and then NanoGlo substrate was added and luciferase levels read by plate reader. B. NT_50_ values of a panel of mouse sera tested with the stable reporter viruses. For ZIKV and YFV NT_50_ tests, the C38 virus was used in both instances. C. Antiviral assay scheme. Huh7 cells (1.5 × 10^4^ cells per well) were seeded the previous day. NITD008 was two-fold serially diluted starting at 10 µM. The dilutions were mixed with virus and plated on the Huh7 cells in a white, opaque 96 well plate and incubated at 37°C for 48 h. Following a 3X PBS wash, results were read by plate reader after addition of NanoGlo substrate. D-E. EC_50_ results from testing NITD008 against the panel of stable reporter viruses in both graphical and table formats, respectively.

One of the aims of this study was to develop stable reporter flaviviruses for neutralization tests and antiviral compound assays. As we have previously done, the stable reporter viruses were used to test a panel of flavivirus-immune mouse sera (Figure 4(A)) in a four-hour neutralization test. NT_50_ results are indicative of the previous infection with the homologous virus yielding the highest NT_50_, though some cross-neutralization by heterologous viruses was observed (Figure 4(B)). Using the flavivirus inhibitor NITD008 [31,32], each virus was used in an antiviral compound assay (Figure 4(C)). Increasing concentrations of NITD008 decreased luciferase expression compared to control (Figure 4(D)) resulting in potent EC_50_ values (Figure 4(E)). These results support our previous data showing that NanoLuc-tagged flaviviruses can be valuable tools in rapid sero-diagnostic assays and antiviral compound screens.

### ZIKV C38 in mosquitoes

**Figure 5.**
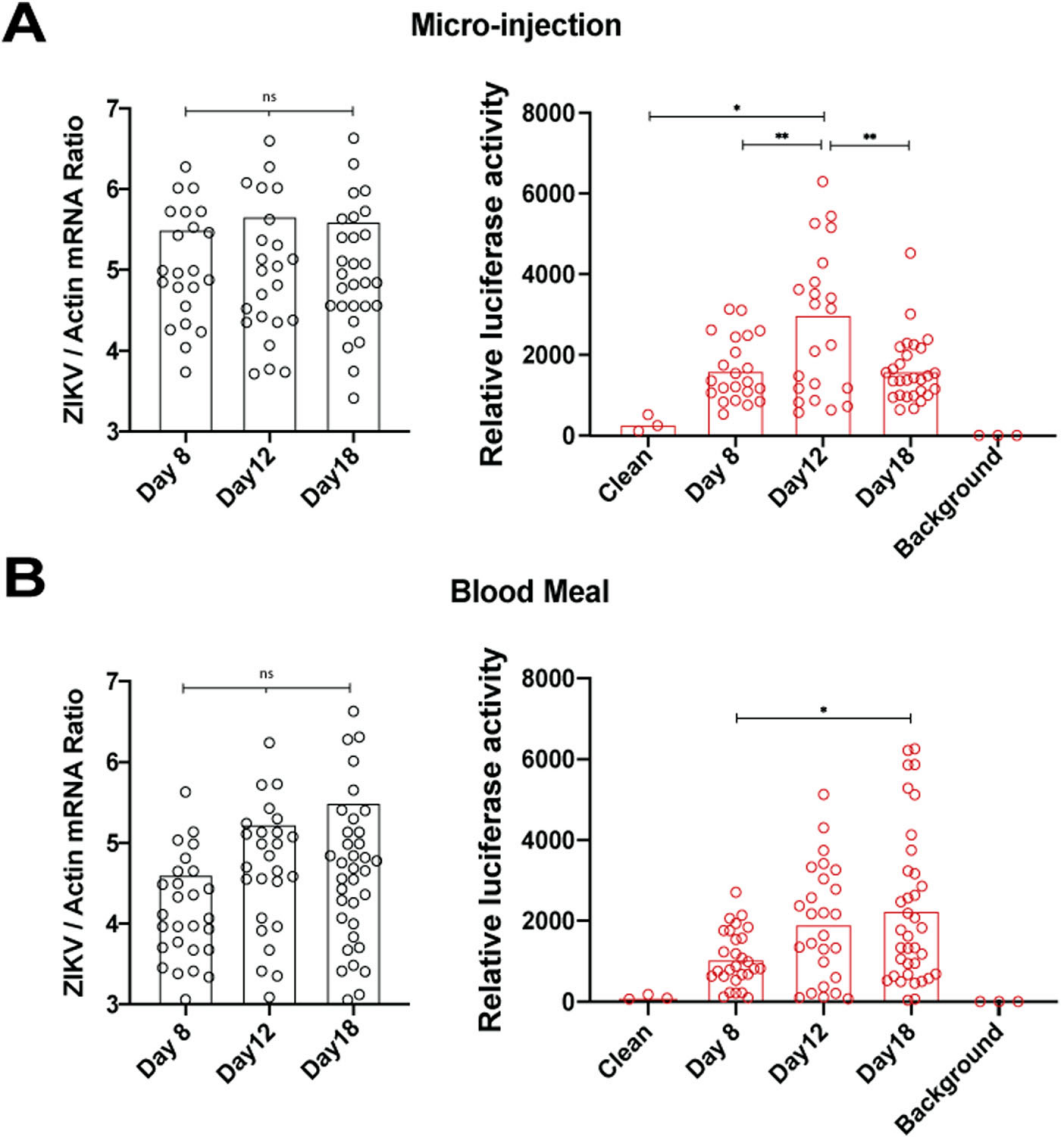
ZIKV C38 Nano in Mosquitoes. A. *Aedes aegypti* mosquitoes were micro-injected with 50 FFU ZIKV C38 Nano (*n* = 30 per day). On days 8, 12, and 18, whole mosquitoes were collected and individually homogenized in PBS. Samples were analyzed by both qPCR (left panel) or luciferase assay (right panel). B. *Aedes aegypti* mosquitoes (n=50 per group) were inoculated by membrane blood-feeding on sheep’s blood spiked with 2 × 10^6^ FFU/mL ZIKV C38 Nano. Mosquitoes were then analyzed as in panel A. For the luciferase assay, clean (uninfected) mosquitoes were used as a control. Values for luciferase activity are reported relative to background (no mosquito) levels. ANOVA with Tukey’s post-hoc test was used to assess significant differences in all panels (*p* > 0.5 = ns, *p* < 0.5 = *, *p* < 0.1 = **, *p* < 0.01 = ***, *p* < 0.001 = ****).

Reporter virus use in mosquito experiments is highly advantageous, where RNA extraction from individual mosquitoes is time-consuming. Intrathoracic injection of mosquitoes with ZIKV C25 showed no viral replication and no luciferase expression (data not shown), possibly due to reporter gene induced replication attenuation. Because ZIKV C38 had more robust luciferase expression compared to C25 in C6/36 cells (data not shown), we characterized ZIKV C38 replication in whole *Aedes aegypti* mosquitoes by both micro-injection, which surpasses the midgut barrier, and membrane blood feeding. Mosquitoes were micro-injected in the thorax with 50 FFU ZIKV C38 and at days 8, 12, and 18, whole mosquitoes were homogenized in PBS and assayed by both qPCR (Figure 5(A), left panel) and luciferase assay (right panel). Although viral RNA did not increase from day 8 to day 12, the luciferase assay shows a statistically significant peak at day 12. In a separate experiment, mosquitoes were allowed to feed on blood spiked with 2×10^6^ FFU/mL ZIKV C38. These mosquitoes were also homogenized on days 8, 12, and 18 and evaluated by both qPCR and luciferase assay (Figure 4(B), right and left panel, respectively). By blood feeding, ZIKV C38 titers increased at each time point by qPCR, though the increase was not statistically significant. Corroboratively, the luciferase activities significantly increased from day 8 to 18. Uninfected mosquitoes were also assayed in Figure 5(B) (right panel) as a negative control. These data show that ZIKV C38 replicates in *Aedes aegypti* mosquitoes and luciferase output can be used to assay viral replication.

## Discussion

Flavivirus reporter constructs have been notoriously unstable since they were first reported [14,33]. Improvements in design [16] have increased the stability, but previous efforts have fallen short of the high standard of ten passages. Here we report a panel of NanoLuc-tagged flaviviruses with stability to at least ten passages in cell culture, which is double the passages routinely reported. It was found that C38 ZIKV and YFV reporter viruses were more stable than their C25 counterparts, and in the case of ZIKV, C38 had a distinct replication advantage. Previous hypotheses for constructing reporter flaviviruses assumes that shorter capsid duplication lengths would be more stable, due to a shorter stretch of homologous sequence. These results challenge that assumption, suggesting that C38 is optimal. The establishment of a stable reporter virus system will greatly facilitate the production of reporter virus in cell culture through viral infection and amplification rather than transfection of viral RNA transcribed from its infectious cDNA plasmid. The ease of stable reporter virus production enables potential high-throughput flavivirus neutralization testing and antiviral screening, as recently demonstrated for reporter SARS-CoV-2 [34,35].

Many different *cis*-acting elements present in the flavivirus capsid coding region have been mapped, including the 5’CS [36], the cHP [37], the 5’ DAR [38], and the DCS-PK [39]. These elements work together to regulate RNA translation, genome cyclization, and viral replication. C25 includes all of those elements except the full DCS-PK, a pseudoknot that has been modeled in various flavivirus genomes, including ZIKV [40], and experimentally been found to aid viral replication in DENV2 [41] and DENV4 [39]. Extending C25 to C38 includes the full DCS-PK, which may explain the increased replication capacity of ZIKV C38 compared to ZIKV C25. We hypothesize that the lack of the DCS-PK in ZIKV C25 caused increased selective pressure and helped drive recombination. Inclusion of DCS-PK in ZIKV C38 lessened this selective pressure, expanding its stability. In contrast, YFV C38 replicated very similarly to YFV C25, supporting a model that YFV lacks, or has a shortened, DCS-PK [39]. Despite this, the lengthened capsid duplication still had a positive effect on YFV stability. It remains to be determined what RNA element in the C38 coding sequence facilitates YFV replication.

Previous work with reporter ZIKV identified C50 as an optimal length for capsid duplication in relation to replication but its effect on stability was not independently tested [18]. In our hands, C38 performed remarkably better in both stability and viral replication when compared to C50. The discrepancy could be due to different ZIKV strains, different sequences flanking the reporter gene, and the absence of a frameshift mutation which was included to help block recombination. ZIKV C50 includes the DCS-PK, along with the other required replication elements and as such would be expected to replicate similar to ZIKV C38. The extra capsid amino acids C39-C50, which contain residues shown to be important in capsid dimerization [42], could allow the C50 capsid fragment to interfere with full-length capsid, thus explaining the attenuation of C50 compared to C38. This selective pressure, along with a larger region for possible recombination, could also be driving the poor stability seen during passaging.

We used sera from known virus-infected mice to demonstrate the utility of the reporter virus for neutralization testing. The reporter virus neutralization assay has been optimized in a 96-well format for high-throughput testing. For clinical use of the reporter neutralization testing, the assay must be first validated using patient sera with well-defined viral infections. The validation could be achieved by comparing the antibody neutralizing titers derived from the conventional plaque reduction neutralization test (PRNT) with those derived from the reporter virus assay. Efforts are ongoing to obtain the well-defined patient sera to perform the clinical validation of reporter virus neutralization assay.

## Conclusion

Together, these results demonstrate that extending the portion of capsid duplicated to make ZIKV and YFV reporter viruses can increase their stability and in the case of ZIKV enhance its replication capabilities in mammalian cells and whole mosquitoes. These data help inform a new generation of stable flavivirus reporter constructs to be used for high-throughput drug screens, serological diagnosis, pathogenesis studies, and transgene delivery.

## Supplementary Material

Supplemental Material
