# Supplementary material for: Identifying optimal capsid duplication length for the stability of reporter flaviviruses
Source: Emerg Microbes Infect. 2020 Oct 14;9(1):2256–65. doi: 10.1080/22221751.2020.1829994 (PMC7594839; doi:10.1080/22221751.2020.1829994)
Supplement: Supplemental Material [file TEMI_A_1829994_SM9710.zip › Supplement Figures/Supplemental Figure captions.docx]

**Supplemental Figure 1.** In vitro ligation scheme for DENV3 (**A**) and DENV4 (**B**). The nucleotide positions of each fragment junction are indicated in red according to GenBank accession numbers EU482459 and FN429920, respectively.

**Supplemental Figure 2. A.** Capsid duplication length of WNV along with IFA images stained with 4G2 and DAPI. **B.** Stability results of passaged WNV-Nano, full length size 1,140 bp

**Supplemental Figure 3. A.** Scheme for passaging reporter viruses. P0 is used to denote viral stock recovered after electroporation. 500 µL of P0 was used to inoculate a T75 flask of confluent Vero cells. Infection was allowed to proceed until cell death was observed. This was repeated until P10 virus was obtained. Viral RNA was obtained from each passage and used in an RT-PCR reaction that bridged the reporter gene. These products were run on an agarose gel to observe band size. **B.** Focus-forming assay using WT ZIKV on Vero cells after a 4-day infection.

**Supplemental Figure 4.** **A.** Growth kinetics comparison of ZIKV C38 and WT ZIKV on Vero cells (MOI 0.01, n=3). Significant differences were assessed with 2-way repeated measures ANOVA with Sidak’s multiple comparisons test (p>0.5=ns, p<0.5=*, p<0.1=**, p<0.01=***, p<0.001=****). Data for ZIKV C38 is the same as shown in Fig 3A.
