# Supplementary figures and images for: Identifying optimal capsid duplication length for the stability of reporter flaviviruses
Source: Emerg Microbes Infect. 2020 Oct 14;9(1):2256–65. doi: 10.1080/22221751.2020.1829994 (PMC7594839; doi:10.1080/22221751.2020.1829994)

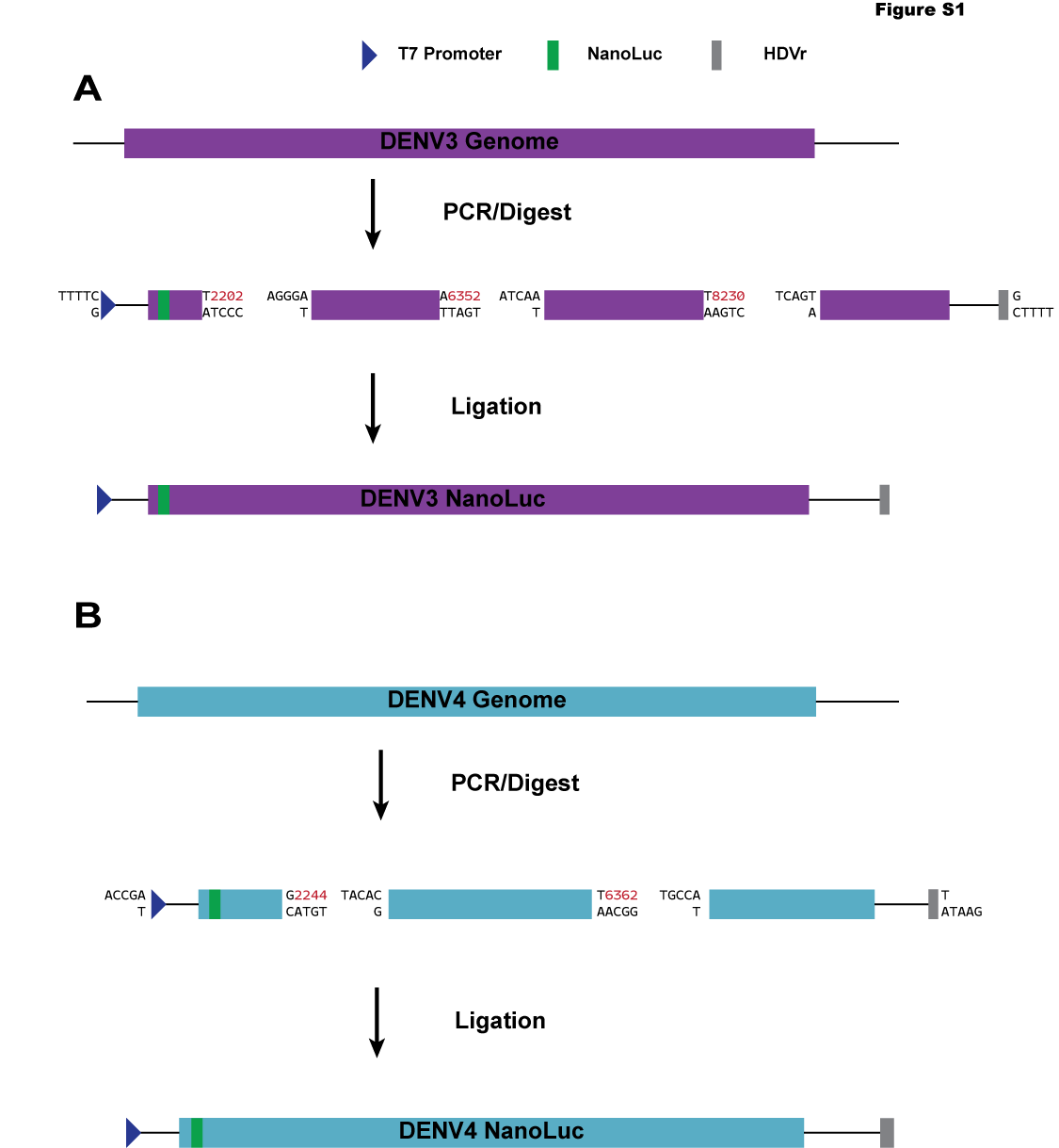

Supplement: Supplemental Material [file TEMI_A_1829994_SM9710.zip › Supplement Figures/FlaviRep Figures-S1.png]

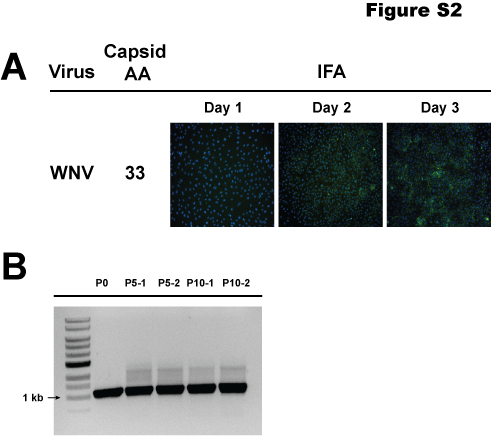

Supplement: Supplemental Material [file TEMI_A_1829994_SM9710.zip › Supplement Figures/Flavirep Figures-S2.png]

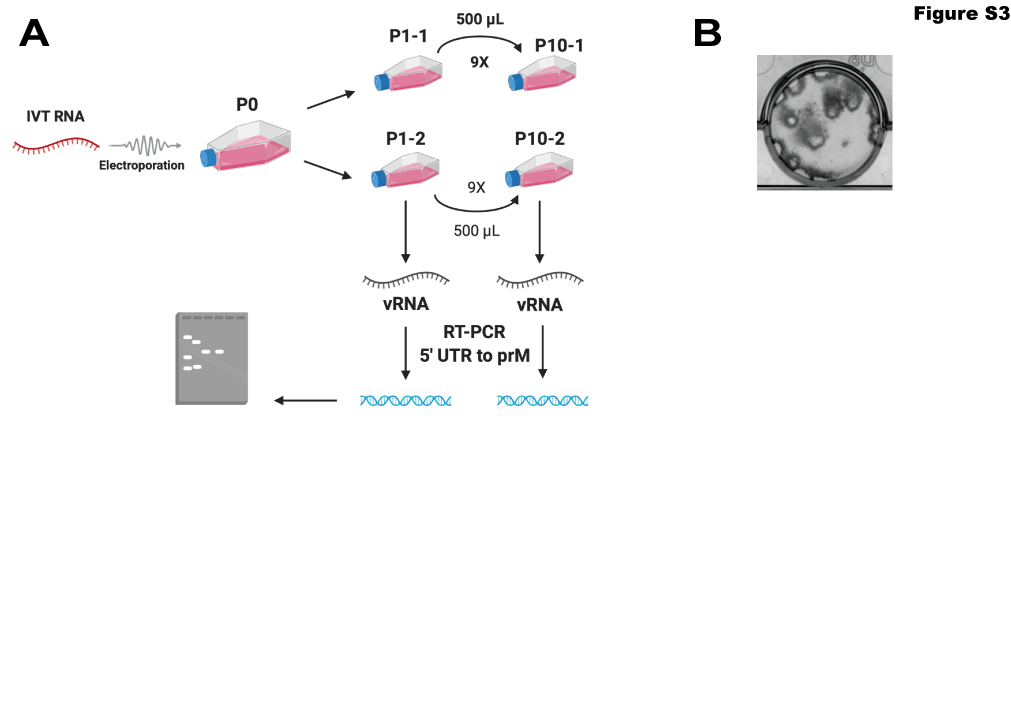

Supplement: Supplemental Material [file TEMI_A_1829994_SM9710.zip › Supplement Figures/FlaviRep Figures-S3.png]

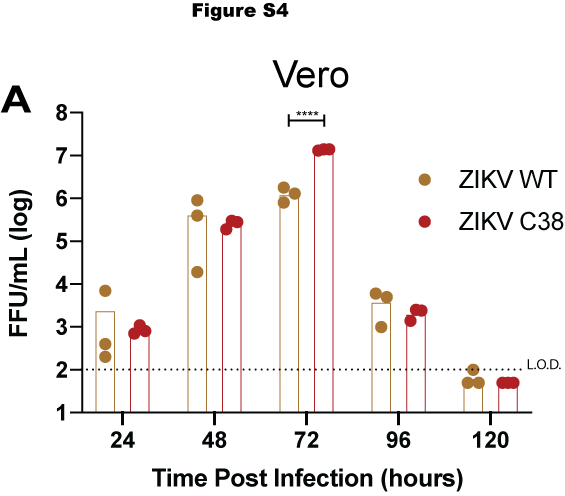

Supplement: Supplemental Material [file TEMI_A_1829994_SM9710.zip › Supplement Figures/Flavirep Figures-S4.png]
